# First molecular characterization of canine parvovirus strains in Sardinia, Italy

**DOI:** 10.1007/s00705-017-3457-3

**Published:** 2017-07-14

**Authors:** S. Dei Giudici, T. Cubeddu, A. Giagu, G. Sanna, S. Rocca, A. Oggiano

**Affiliations:** 1 Istituto Zooprofilattico Sperimentale della Sardegna G. Pegreffi, Via Vienna 2, 07100 Sassari, Italy; 2 Dipartimento di Medicina Veterinaria, Università di Sassari, Via Vienna 2, 07100 Sassari, Italy

**Keywords:** Canine parvovirus (CPV), Phylogenetic analysis, VP2 gene

## Abstract

Canine parvovirus type 2 (CPV-2) is responsible of acute hemorrhagic gastroenteritis in young dogs. CPV-2 emerged in 1978 in the USA, but new antigenic types, CPV-2a, 2b and 2c, have completely replaced the original type. In this study, we analyzed 81 animals collected in Sardinia, Italy. The VP2 sequence analysis of 27 positive samples showed that all antigenic CPV-2 types are circulating. CPV-2b seems to be the most widespread variant, followed by CPV-2a. Furthermore, 12 CPV-2b strains displayed further amino acid substitutions and formed a separate cluster in a phylogenetic tree, indicating regional genetic variation.

Canine parvovirus type 2 (CPV-2) is a small, non-enveloped, single-stranded DNA virus with a diameter of 18 to 26 nm, belonging to the genus *Protoparvovirus* [10]. The genome is approximately 5.2 kb long and has two open reading frames (ORF), encoding two non-structural proteins (NS1, NS2) and two capsid proteins (VP1, VP2) [29]. The substitution of few amino acids in VP2, the main viral protein of the icosahedral capsid, alters relevant biological characteristics of the virus [25].

The virus, named CPV-2 to distinguish it from the antigenically unrelated minute virus of canines (MVC or CPV-1) [3, 23], spread in 1978 and caused epizootic gastroenteritis in dogs [6]. In 1979 and 1984, two new antigenic types, named CPV-2a and 2b, respectively, replaced the original CPV-2 worldwide [24, 26], gaining the ability to replicate efficiently in cats. The new antigenic types, CPV-2a and CPV-2b, differ from the original CPV-2 at least in five or six amino acids of the VP2 capsid protein. An additional amino acid change at position 297 (Ser → Ala), both in CPV-2a and CPV-2b, has lead to the designation “new CPV-2a” and “new CPV-2b” [10]. Moreover, in 2000 another antigenic variant, characterized by the amino acid substitution 426-Asp → Glu was reported [4]. This variant, designed CPV-2c, was subsequently reported all over the world [10] and has been associated with a severe clinical course with higher mortality rates [8]. The amino acids 426 and 297 of the VP2 are spatially close to residues 97 and 300, respectively, of the threefold spike domain on the capsid surface [15]. Structural studies have shown that this region interacts with the transferrin receptor (TFR) of the host cell to mediate infection, defines the host range, and is highly antigenic [1, 21, 22].

Most of the commercial vaccines currently available worldwide are based on CPV-2 or CPV-2b, and several studies have demonstrated that they are able to cross-protect against all four antigenic types, including the newly emerged CPV-2c [33].

The aim of the study was to characterize for the first time the CPV strains circulating in Sardinia (Italy), by molecular analysis. The partial VP2-encoding gene from 27 strains was amplified by PCR and sequenced. The characterization was performed by phylogenetic analysis of the sequences and amino acid composition of the different CPV-2 strains.

We analyzed 93 samples collected from 81 animals in the period 2005 to 2013, mainly in northern Sardinia. In detail, we analyzed 20 fecal samples from adult dogs with suspected parvovirosis, 55 samples (gut and mesenteric lymph nodes) collected from 12 adult cats and 43 dogs that had died from natural causes and did not have clinical signs of CPV, and 18 different tissue samples (gut, brain and spleen) collected from six carcasses of puppies with clinical signs of parvovirosis. DNA extraction was performed using a DNeasy Blood & Tissue Kit (QIAGEN), according to the manufacturer’s instructions. The presence of CPV-2 strains was detected using conventional polymerase chain reaction (cPCR) as described by Senda et al. [31]. To investigate the possible presence of viral coinfections, fecal and gut samples were also analyzed for the detection of canine or feline coronavirus (Cov) RNA as described previously [12], and brain and spleen samples were analyzed for the detection of canine distemper virus (CDV) RNA [14]. Characterization of viruses in the CPV-2-positive samples was performed by amplification and direct sequencing of the hypervariable region (611 bp long) of the gene encoding VP2, using primers Hfor and Hrev as described previously [4]. Our sequences were aligned using ClustalW software with canine parvovirus sequences retrieved from GenBank. Sequence translation and the phylogenetic analysis were performed using MEGA 6.0 software [34]. Phylogeny was estimated by the maximum likelihood (ML) algorithm with the HKY+I model. The reliability of the trees was calculated using 1000 bootstrap replicates. The evolutionary model that best fitted the data was selected using JmodelTest [28].

Out of 81 animals screened by conventional PCR assay, 27 (25 dogs and 2 cats) were positive for CPV-2 (31.4%). Five of the positive samples were fecal samples, and 22 were tissue samples. The sample identification number, source, host, and collection year are reported in Table 1. All samples tested PCR negative for CDV, and one gut sample tested COV positive. Five of the six puppies with clinical signs of parvovirosis tested CPV-2 positive in all of the tissues (gut, brain and spleen) analyzed, but only brain tissues were used for molecular characterization. Sequences of the CPV-2 positive samples were 99 to 100% identical to the VP2 sequences of other canine parvovirus strains available in GenBank. Our sequences were deposited in the GenBank database, with the accession numbers KM262057 to KM262083 (Table 1). Phylogenetic analysis revealed that all CPV variants are present in Sardinia (Fig. 1). Specifically, nine strains were identified as CPV-2a, 16 as CPV-2b, and two as CPV-2c. A amino acid sequence analysis (Table 1) showed that nine samples have Ala at aa position 297 and Asn at position 426, which is characteristic of new CPV-2a strains; 15 contained Ala at aa position 297 and Asp at position 426, which is characteristic of new CPV-2b; one had Ser at aa position 297 and Asp at position 426, which is characteristic of CPV-2b (Sar48), and two had Glu at aa position 426, which is characteristic of CPV-2c. The sequence of strain Sar9 contained the amino acid Lys instead of Asn at position 320. In addition, 12 of the 15 new CPV-2b isolates, including one from a cat, contained further amino acid substitutions, 371Ala→Gly and 418Ile → Thr. The strains Sar54 andSar55 also had a third amino acid substitution, 440Thr → Pro. In the phylogenetic tree, these strains formed a distinct cluster with good statistical support (bootstrap value, 87), together with the Italian strain KF373599, which was isolated in Sicily. Sar54 and Sar55 were detached from this cluster, forming a separate one. Out of two PCR-positive cats, one was infected by a new CPV-2a strain, and the other by a new CPV-2b strain.

**Table 1:** Sample characteristics, amino acid mutations in the VP2 sequence, GenBank accession numbers and classification of CPV2 strains analyzed in this study

Sample	Source	Host	Year	Amino acid position								Accession number	CPV variant
				aa 297	aa 300	aa 305	aa 321	aa 371	aa 418	aa 426	aa 440		
SAR1	Feces	Canis familiaris	2005	Ala (A)	Gly (G)	Tyr (Y)	Asn (N)	Ala (A)	Ile (I)	Asp (D)	Thr (T)	KM262057	New CPV-2b
SAR2	Feces	Canis familiaris	2006	.	.	.	.	.	.	Asn (N)	.	KM262058	New CPV-2a
SAR3	Feces	Canis familiaris	2006	.	.	.	.	.	.	Glu (E)	.	KM262059	CPV-2c
SAR4	Feces	Canis familiaris	2006	.	.	.	.	.	.	Asn (N)	.	KM262060	New CPV-2a
SAR5	Feces	Canis familiaris	2007	.	.	.	.	.	.	Asp (D)	.	KM262061	New CPV-2b
SAR9	Gut	Canis familiaris	2012	.	.	.	Lys (K)	.	.	Asp (D)	.	KM262062	New CPV-2b
SAR10	Gut	Canis familiaris	2012	.	.	.	.	.	.	Asn (N)	.	KM262063	New CPV-2a
SAR11	Gut	Canis familiaris	2012	.	.	.	.	.	.	Glu (E)	.	KM262064	CPV-2c
SAR13	Gut	Canis familiaris	2012	.	.	.	.	.	.	Asn (N)	.	KM262065	New CPV-2a
SAR23	Gut	Canis familiaris	2012	.	.	.	.	Gly (G)	Thr (T)	Asp (D)	.	KM262066	New CPV-2b
SAR24	Gut	Canis familiaris	2012	.	.	.	.	Gly (G)	Thr (T)	Asp (D)	.	KM262067	New CPV-2b
SAR25	Gut	Felis catus	2012	.	.	.	.	Gly (G)	Thr (T)	Asp (D)	.	KM262068	New CPV-2b
SAR33	Gut	Canis familiaris	2012	.	.	.	.	.	.	Asn (N)	.	KM262069	New CPV-2a
SAR39	Gut	Canis familiaris	2012	.	.	.	.	.	.	Asn (N)	.	KM262070	New CPV-2a
SAR40	Gut	Canis familiaris	2012	.	.	.	.	.	.	Asn (N)	.	KM262071	New CPV-2a
SAR41	Gut	Felis catus	2012	.	.	.	.	.	.	Asn (N)	.	KM262072	New CPV-2a
SAR42	Gut	Canis familiaris	2012	.	.	.	.	.	.	Asn (N)	.	KM262073	New CPV-2a
SAR43	Gut	Canis familiaris	2012	.	.	.	.	Gly (G)	Thr (T)	Asp (D)	.	KM262074	New CPV-2b
SAR45	Gut	Canis familiaris	2012	.	.	.	.	Gly (G)	Thr (T)	Asp (D)	.	KM262075	New CPV-2b
SAR48	Gut	Canis familiaris	2013	Ser (S)	.	.	.	.	.	Asp (D)	.	KM262076	CPV-2b
SAR49	Gut	Canis familiaris	2013	.	.	.	.	Gly (G)	Thr (T)	Asp (D)	.	KM262077	New CPV-2b
SAR50	Gut	Canis familiaris	2013	.	.	.	.	Gly (G)	Thr (T)	Asp (D)	.	KM262078	New CPV-2b
SAR51	Brain	Canis familiaris	2013	.	.	.	.	Gly (G)	Thr (T)	Asp (D)	.	KM262079	New CPV-2b
SAR52	Brain	Canis familiaris	2013	.	.	.	.	Gly (G)	Thr (T)	Asp (D)	.	KM262080	New CPV-2b
SAR53	Brain	Canis familiaris	2013	.	.	.	.	Gly (G)	Thr (T)	Asp (D)	.	KM262081	New CPV-2b
SAR54	Brain	Canis familiaris	2013	.	.	.	.	Gly (G)	Thr (T)	Asp (D)	Pro (P)	KM262082	New CPV-2b
SAR55	Brain	Canis familiaris	2013	.	.	.	.	Gly (G)	Thr (T)	Asp (D)	Pro (P)	KM262083	New CPV-2b

**Fig. 1:**
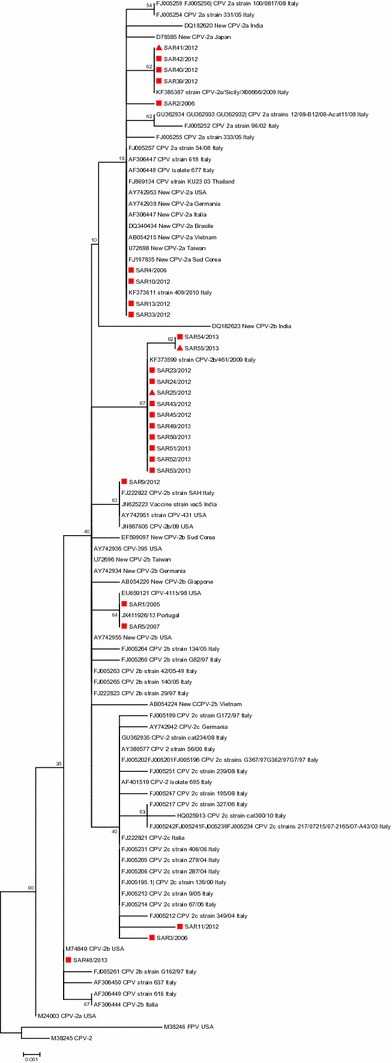
Phylogenetic tree of the 27 CPV2 strains in our study based on a 587-bp-long portion of the VP2 sequence. MEGA 6 was used to construct a maximum-likelihood tree based on the HKY+I model. The reliability of the tree was assessed by 1000 bootstrap replications. The reference sequences were retrieved from the GenBank database and were from Italy (KF385387, KF373611/13, AF306447, AF306448, AF306447, FJ005257, FJ005255, GU362934, GU362933, GU362932, FJ005252, FJ005259, FJ005256, FJ005254, KF373599, FJ222822, FJ005263, FJ005265, FJ222823, FJ005199, GU362935, AY380577, FJ005251, FJ005202, FJ005201, FJ005196, FJ005247, AF401519, HQ025913, FJ005217, FJ005242, FJ005241, FJ005239, FJ005234, FJ222821, FJ005231, FJ005205, FJ005206, FJ005212, FJ005214, FJ005213, FJ005195, FJ005261, AF306450, AF306449, AF306444) and from various other parts of the world (Oriental strains: FJ197835, U72698, AB054215, FJ869134, DQ182620, DQ182623, JN625223, D78585, EF599097, U72696, AB054220, AB054224; European strains: AY742938, AY742934, JX411926, AY742942; American strains: DQ340434, AY742953, AY742951, JN867605, AY742936, EU659121, AY742955, M74849, M24003, M38245, and FPV 38246). CPV-2 types and countries are indicated after the accession number. The canine isolates analyzed in this study are indicated by a red square, and the feline isolates are indicated by a red triangle (color figure online)

Our data revealed that the new CPV-2b is the most widespread variant, followed by the new CPV-2a strain. The CPV-2c, despite being present in Sardinia since at least 2006 (Sar4), is the least common variant. This suggests that the CPV-2c strain has not spread at the same rate in Sardinia and has not taken over, even temporarily, as has been described for other geographical areas [9]. Only one sample contained the CPV-2b old variant (Ser297), but it was probably a new entry because the sample was collected in 2013 (Sar48). These data showed that all known variants are circulating in Sardinia. The prevalence of the new CPV-2b in Sardinia is in contrast with findings in other regions of Italy, where the prevalent strain is CPV-2c, followed by CPV-2a [7]. Phylogenetic analysis showed that the Sardinian strains were closely related to Italian isolates, with the exception of Sar1 and Sar5, which clustered with isolates from the USA and Portugal (Fig. 1), and Sar48, which was 100% identical to strain M74849 from the USA.

CPV has a worldwide distribution. CPV-2c has been found mainly in South American and European countries [19]. In Africa, the Middle East, Asia and Australia, CPV-2b and CPV-2a are the predominant variants, while CPV-2c seems to be absent [11, 13, 17, 32, 36, 37]. In Europe, there seems to be a more equal spreading of the three variants [10, 19], which could be related to geographical position and a different commercial flows of dogs. The 12 Sardinian CPV-2b strains, which formed a single separate clade in the phylogenetic tree, are characterized by the substitutions I418T and A371G, both of which are located in the GH insertion of VP2 [5]. This region includes the amino acids 267-498 between the βG and βH strands, is responsible for creating the 22-Å-long spikes around the icosahedral three-fold axes on the surface of the capsid, and shows the greatest variability among parvoviruses [5]. The I418T mutation was previously reported in both CPV-2a and CPV-2b isolates from Italy [2, 4, 8] and in CPV-2a isolates from Korea [16]. The A371G substitution, which is caused by a C → G transversion, is present in only one GenBank entry, the Italian sequence KF373599, for which there is no literature reference. In addition to the above-mentioned mutations, the strains Sar54 and Sar55 also displayed the substitution, T440P, which is caused by an A → C transversion. Position 440 is considered to be the main antigenic site of the virus, being located at the top of the threefold spike. Another mutation at this position, T440A, has been reported, detected for the first time in Italy by Battilani et al. [2] and also found in Korea [16], Uruguay [18], China [35] India [20] and Thailand [27]. The substitution T440P, detected in our strains, has never been reported so far. It is noteworthy that five of twelve samples belonging to this cluster were from brains of puppies with clinical signs of parvovirosis. As already reported, CPV can be detected in the brain in experimental and natural infections [9, 30]. The 12 CPV-2b strains account for about 44% of those examined, and they formed a separate cluster in the phylogenetic tree, indicating regional genetic variation of CPV-2 strains [17]. Our results showed that all currently known strains of CPV are circulating in northern Sardinia but that the new CPV-2b is the more widespread variant. However, because most of the samples were from northern Sardinia, the data do not necessarily reflect the overall distribution of CPV variants in Sardinia. Further extensive epidemiological investigation will be necessary to verify these results.

The finding of new or rare amino acid mutations confirms that this virus is continuously evolving. Although the available vaccines seem to cross-protect against all CPV-2 types, the spread of new variants could be responsible of immunity failure, underlining the need to carefully and constantly evaluate this virus through molecular epidemiology studies in order to prevent and control CPV-2 infection.
